# Remediation Program with Working Memory and Reading for Students with Learning Difficulties: Elaboration and Pilot Study

**DOI:** 10.3390/children12040426

**Published:** 2025-03-28

**Authors:** Isabella Nicolete Xavier, Simone Aparecida Capellini

**Affiliations:** 1 Post-Graduation Program in Speech-Language Pathology, Faculty of Philosophy and Sciences, São Paulo State University “Júlio de Mesquita Filho”, Marília 17525900, Brazil; 2 Department of Speech-Language Pathology, Faculty of Philosophy and Sciences, São Paulo State University “Júlio de Mesquita Filho”, Marília 17525900, Brazil; sacap@uol.com.br

**Keywords:** learning, reading development difficulties, working memory, reading remediation, reading skills, intervention study

## Abstract

Background/objectives: A child’s working memory needs to be efficient in order to perform well at school, because its manipulative function needs to work properly in order to compose and decompose words, a skill that is necessary for reading. Therefore, if a child with an alteration in this type of memory reads a more complex sentence, they will have difficulty storing it until other cognitive processes involved in language comprehension and production take place, leading to impaired reading comprehension. The aim of this study was to develop and verify the applicability of a remediation program for working memory and reading in students with learning difficulties from the third to fifth grades of primary school. Methods: The study was carried out in two phases: phase 1 developed the program on the basis of a literature review, and phase 2 verified the applicability of the program in a pilot study with 21 schoolchildren divided into two groups. The subjects were subjected to tests of metalinguistic and reading skills and the Brief Child Neuropsychological Assessment Instrument. Results: The working memory and reading remediation program consisted of 11 tasks developing phonological and visuospatial working memory. From the results of the application of the Remediation Program With Working Memory and Reading (RP-WMR) in a pilot study, it was possible to verify the applicability of the program; in other words, the strategies developed for students with learning difficulties can be generalised and applied to students who have deficits in working memory and reading. Conclusions: The result of this research indicates that the structured program for remediation of working memory difficulties has proven to be applicable and can help education professionals as a tool for intervening in working memory deficits and reading decoding skills presented by students with learning difficulties.

## 1. Introduction

Reading development is a complex process that involves multiple cognitive and linguistic factors, including working memory [1]. The terms “working memory” (WM) and “operational memory” (OM) are used to refer to a mental operation that has the ability to hold and manipulate information for brief periods during demanding cognitive activities [2,3].

According to the model proposed by Baddeley and Hitch [4], WM can be divided into four subsystems: a. the central executive, a limited-capacity processing system at the centre of this WM model, responsible for regulating the flow of information, coordinating access to and retrieval of more durable knowledge, such as long-term memory, and controlling action and the programming of various cognitive activities. Its mediating role includes coordinating the activity of both the phonological loop and the visuospatial sketchpad. Additionally, the central executive is functionally responsible for regulating three key cognitive processes: shifting, updating, and inhibition; b. the phonological loop, a temporary storage mechanism capable of storing limited amounts of verbal and acoustic information with the active support of vocal and subvocal rehearsal; c. the visuospatial sketchpad, which represents the information according to its visuospatial characteristics [5,6]; and d. the episodic buffer, which was identified later and is believed to be responsible for integrating information from a variety of sources in the cognitive system, including temporary and long-term memory systems [4,5,7,8].

Previous research has demonstrated a strong relationship between the central executive and the phonological loop components of WM, highlighting their crucial role in acquiring new knowledge and developing complex skills [3,7,9,10]. The phonological subcomponent of WM seems to play an important role in learning the phonological structure and representation of letters and new words, especially at the beginning of reading development, when the student is developing the predictive skills needed for literacy competence. It is crucial to attain a more comprehensive comprehension of the degree to which working memory (WM) is associated with reading performance, as well as the factors that influence this association, which have exhibited significant variability, as some studies have found a minimal impact of WM on reading [11], while other studies have demonstrated WM nearly or entirely accounts for reading performance [12]. From a developmental perspective, the research on how WM is involved in different reading skills has addressed two general cognitive theories: the Intrinsic Cognitive Load Theory, which posits that the relation between reading and WM can be mostly affected by the complexity of reading tasks [13,14], and the Dual Process Theory, which postulates that reading experience is influenced by the degree of WM involvement in reading [15]. Therefore, for a child to perform in school, WM must be efficient, and its manipulative function is needed to compose and decompose words, a skill necessary for reading [16,17].

A child in the literacy phase with alterations in this type of memory may have difficulty decoding and storing more complex sentences until other cognitive processes involved in language comprehension and production develop, thus causing impairments in reading comprehension and its subsequent reproduction [18,19].

Because students with WM deficits have difficulties retaining information that allows them to complete a task [20,21], they exhibit differences in reading both regular and irregular words, prioritising the phonological approach, i.e., the grapheme—phoneme correspondence process. This approach results in lower performance in decoding and phonological WM tests due to an overload caused by this route, as well as in the reading fluency task, thus affecting reading and writing development [22,23].

On the basis of the considerations described in the literature above, it is possible to conclude that low WM capacity may be a risk factor for low academic performance and, consequently, for educational differences. There is increasing evidence of deficits in WM in schoolchildren with learning difficulties, learning disorders, mathematical difficulties, attention-deficit/hyperactivity disorder (ADHD), and even motor difficulties [10,24].

Recognising the importance of WM in reading, as well as the development of reading in WM, highlights the need for support and teaching strategies that help children develop this skill and competence together in a two-way manner. Thus, the hypothesis in the literature is that working memory and decoding skills can be trained, which will positively impact reading and writing activities in the classroom context [24,25,26].

On the basis of this hypothesis, increasing efforts have been directed towards the development of effective WM interventions. Although there is no consensus in the literature regarding the effectiveness of training WM [27,28], studies in schoolchildren with learning difficulties and/or disorders who exhibit changes in WM have shown that these students show improvements in reading and writing after specific WM training [19,29].

In this regard, the relationship between WM intervention and reading development is not uniform and may vary depending on multiple factors. These factors include the differential impact of training in distinct WM domains on specific academic skills, the importance of aligning WM training with academic demands, the potential moderating effects of children’s individual characteristics and training parameters on intervention efficacy, and the implications of implementing cognitive training in isolation or in combination with academic interventions to optimise skill transfer [9,16,25,26,27,28].

However, some studies have investigated key aspects of WM training, including its suitability for different domains (verbal, numerical, or visuospatial) based on targeted academic skills, the impact of its academic focus (e.g., comprehension vs. decoding), the influence of child-related and training-specific factors (e.g., age, intensity, duration) on effectiveness, and whether combining cognitive training with academic interventions enhances skill transfer [30,31,32,33,34].

Thus, a program combining WM strategies and the reading decoding skill can improve our understanding of how and to what extent these two cognitive–linguistic skills, when stimulated together, can influence the development of the decoding skill, as well as the use of the memory generative mechanism for the composition of words and learning new words.

In view of the above background, this study hypothesises that a remediation program based on the use of the generative memory mechanism, i.e., using strategies involving phonological and visuospatial WM and reading, may contribute to the development of the decoding skill, a type of memory that is necessary for reading words and learning new words through the use of sounds and syllables in different word positions, in students with learning difficulties. Thus, the aim of this study was to develop and verify the applicability of a remediation program for difficulties in phonological and visuospatial WM in third to fifth grade students with learning difficulties in a pilot study.

## 2. Materials and Methods

This study was conducted with the approval of the Research Ethics Committee of the Universidade Estadual Paulista (UNESP) “Júlio de Mesquita Filho”, Faculty of Philosophy and Sciences, Marília, São Paulo, under protocol number 67680623.9.0000.5406.

To provide a clearer understanding, the methodological design used in this study is presented in two phases: phase 1 focuses on the development of the Remediation Program with WM and Reading (RP-WMR), called “Programa de Remediação com a Memória Operacional e Leitura” (PR-MOL) in Brazilian Portuguese, in students with learning difficulties based on a review of the literature, and phase 2 verifies the applicability of the program developed in phase 1 in a pilot study.

### 2.1. Phase 1: Development of the PR-MOL

To develop the PR-MOL, a literature review was performed focusing on the following information: (1) the tasks contained in the training programs for WM; (2) descriptions of the tasks and their objectives; (3) the duration of the tasks; and (4) the number of sessions.

The literature review was performed by searching national and international databases, including Scielo, PubMed, Science Direct, and Procast, which are available online.

The literature review period lasted 9 months, between March and December 2023, focusing on studies published in the last 15 years. The selection criteria prioritised peer-reviewed articles, especially those that presented empirical data and systematic reviews. For the search in each database, search strategies included specific descriptors in English (working memory, training, intervention, children, students, reading skills, reading, reading disorders, reading difficulties) and in Portuguese (memória operacional, programa de treinamento, programa de intervenção, escolares, habilidades de leitura, transtornos de aprendizagem, dificuldades de leitura, dificuldade de aprendizagem). The Boolean operators used to effectively connect the descriptors were: (“working memory”) AND (“training” OR “intervention”) AND (“children OR “students” OR “schoolchildren”) AND (“reading skills” OR “reading” OR “reading disorders” OR “reading difficulties”).

With these search strategies, 69 articles were identified in the national and international databases. After reading the titles and abstracts, 38 articles were excluded. The remaining 31 articles were read in full, and the studies were assessed with the inclusion and exclusion criteria, identifying a final number of 18 articles that were effectively analysed in this study. Both authors were involved in the screening of titles and abstracts. A dual screening process was conducted, where each author independently reviewed all identified studies to ensure consistency and reduce bias. In cases of disagreement or uncertainty regarding the application of inclusion/exclusion criteria, discussions were held to reach a consensus.

Regarding the inclusion/exclusion criteria, articles related to the research topic and matching the search descriptors were selected. Studies were included if they focused on children or students, specifically those aged 4–11 years, and involved interventions or training programs aimed at improving working memory, particularly in the context of reading skills, reading disorders, or reading difficulties. Articles that did not meet these criteria were excluded, including those that focused on populations outside the scope of the review, such as the elderly or individuals with intellectual disabilities. Additionally, studies that did not directly address working memory, training interventions, or reading-related skills were excluded. Articles that were not fully available for review were also excluded from the study.

The tasks used for phonological and visuospatial WM training in the included studies are outlined in Table 1.

In the included studies, the tasks used for phonological WM training were repetition of words, pseudowords, digits, and colours in the same order and/or in reverse order; recall of sequences of phonological representations of graphemes and comparisons between them; naming of figures and digits; execution of simultaneous tasks to perform in a requested order; memorisation of short stories; n-back tasks, in which the recall of one of the elements of the presented sequence is requested according to its position; and interpretation of sentences and memorisation of the last word of each of the sentences. For visuospatial memory, the tasks included immediate repetition of a sequence of visuospatial information in the same order, tracing of locations presented sequentially by a target stimulus, replication of the visuospatial path in a maze, identification and retrieval of hidden objects, retelling of films and stories, memory games, and mental rotation of letters, among others.

Of all the articles analysed, 14 reported a significant improvement in performance on WM tasks and, consequently, an improvement in reading development for both students with learning difficulties and those with learning disorders [19,24,35,36,37,38,39,40,41,42,43,44,45,46]. Improvements were observed in decoding tasks, fluency, and especially reading comprehension. These results are similar to those of previous studies [2,6] that emphasised the importance of WM in the academic success of schoolchildren.

#### Selection of Stimuli for the PR-MOL for Students with Learning Difficulties

On the basis of the literature review described above, the remediation program was designed with tasks using linguistic stimuli (real words and pseudowords) and visual stimuli (pictures). The real words were selected from the word bank of E-reading II [47], which is divided into high-, medium-, and low-frequency words on the basis of the frequency of occurrence of the words in elementary school textbooks. A balanced selection of low-, medium-, and high-frequency words was included. The criteria used to balance the selection of words were based on their syllabic structure to match the different syllabic patterns in the word sequence tasks. Subsequently, this approach ensured that the amount of information that would be processed and manipulated in working memory was balanced by adjusting the syllabic complexity of each word depending on the number of words inserted in the word sequences, without overloading memory. In particular, nouns were selected because of the possibility of being represented by pictures.

The program consisted of a total of 173 words and 262 pseudowords. The real words included 16 monosyllabic, 68 disyllabic, 56 trisyllabic, and 10 polysyllabic words. Owing to the low variability in polysyllabic words in the word bank, 23 polysyllabic words with four and five syllables, taken from the Michaelis online dictionary, were added, for a total of 33 polysyllabic words.

The real words and their respective images were printed on rectangular cards, with the word written in capital letters, lowercase letters, and cursive on one side and the representative picture on the other side.

The PR-MOL consisted of 11 tasks divided into two modules for administration in 10 sessions of 1 h per session. Module 1 included tasks involving phonological WM, and Module 2 included tasks involving visuospatial working memory.

The eleven tasks in the program were cumulative, i.e., they were designed to be performed in the ten program sessions, but in each session, different stimuli were used. In odd-numbered sessions (1, 3, 5, 7, 9), tasks 1 to 6, focusing on phonological WM, were performed, whereas in even-numbered sessions (2, 4, 6, 8, 10), tasks 7 to 11, focusing on visuospatial WM, were performed.

In all the tasks, the student was presented with sequences of stimuli (words or pseudowords) that gradually increased from two to six stimuli according to the storage capacity of WM [48].

Each of the sequences followed a pattern of syllabic extension of the stimuli, as shown below:

1. Two stimuli: a monosyllabic word and a disyllabic word
2. Three stimuli: a monosyllabic word, a disyllabic word, and a trisyllabic word
3. Four stimuli: a monosyllabic word, a disyllabic word, a trisyllabic word, and a polysyllabic word
4. Five stimuli: a disyllabic word, a trisyllabic word, and three polysyllabic words
5. Six stimuli: two trisyllabic words and four polysyllabic words.

To promote the students’ interest in performing the tasks, a playful context was designed with a reward strategy named “Assembling the Memory Treasure”, with the students drawing coins for each task. In addition to the boards used to perform the tasks, a board with a city map, cards for drawing coins and places in the city, personalised coins in a chest, and a board to fill with the coins were prepared.

The PR-MOL was composed of two modules, as shown in Table 2.

### 2.2. Phase 2: Applicability of the PR-MOL in a Pilot Study

#### 2.2.1. Participants

Participants in this study were selected from a municipal public school in a city in the interior of the state of São Paulo with a lower-middle socioeconomic level that follows the global literacy program. Initially, the students who had learning difficulties were indicated by the teachers, and the school records were consulted to verify whether the students met the established inclusion and exclusion criteria, described below.

Inclusion criteria:

- Presence of learning difficulties confirmed by standardised assessment procedures (lower performance of students in the metalinguistic and reading skills test [PROHMELE]).

Exclusion criteria:

- Previous participation in speech therapy, pedagogical, or psychopedagogical intervention programs.
- Presence of genetic syndromes, intellectual disability, or ADHD, as described in the school records.

#### 2.2.2. Sample Characterisation

This study was a blind, quasi-experimental, cross-sectional study conducted with a convenience sample of students with learning difficulties.

A total of 24 schoolchildren of both sexes, aged between 8 years and 11 years and 11 months, from the third, fourth, and fifth years of elementary school I were selected to participate in phase 2 of this study according to the inclusion and exclusion criteria. Three of the students were excluded because they changed schools. The students were divided into two groups:

- Group I (GI): Eleven elementary school students with learning difficulties, consisting of nine males and two females aged between 8 years and 11 years and 11 months, with four students from the third year, three students from the fourth year, and four students from the fifth year, underwent the PR-MOL and post-testing.
- Group II (GII): Ten elementary school students with learning difficulties, consisting of four males and six females from the third to fifth grades, were paired according to age group and education level with the students from GI and did not undergo the PR-MOL.

#### 2.2.3. Instruments

Pre- and post-testing were performed with the PROHMELE assessment [49] and the Brief Child Neuropsychological Assessment Instrument NEUPSILIN-Inf [50], as described below.

The PROHMELE assessment is used to evaluate metalinguistic and reading skills. This assessment is composed of fifteen syllabic and phonemic identification and manipulation tests, a pseudoword repetition test, a real-word reading test composed of 133 words, and a pseudoword reading test consisting of 27 pseudowords, which cover context-independent graphophonemic correspondence rules (Rule D1), context-dependent graphophonemic correspondence rules (Rule D2), and the rule of values of the letter “X” exclusively dependent on the mental and orthographic lexicon (Rule D4). The results were corrected and analysed following the instructions and regulations of the protocol manual.

The NEUPSILIN-Inf is a brief neuropsychological assessment tool that assesses orientation, attention, visual perception, memory, arithmetic skills, language, visuoconstructive skills, and executive functions. To evaluate phonological WM, the Repetition of Digit Sequence in Indirect Order (reverse order) and Pseudoword Span tests were used, each composed of eight sequences. To evaluate visuospatial WM, the Corsi block test was used. Both instruments used were validated for content validity (test specifications and construction process with analysis of agreement between judges), construct validity (developmental changes, investigated by means and standard deviations, observed in each age group, validity of internal structure through confirmatory factor analysis), and criterion validity to differentiate children with and without difficulties in the skills assessed. The results were corrected and analysed according to the instructions in the instrument manual.

#### 2.2.4. Procedures

All the subjects who participated in this study completed the pre- and post-testing evaluations in a large, quiet room without distractions. The study was conducted during the three-month period from March 2023 to June 2023 in a public school of the municipal elementary school network located in the interior of the state of São Paulo.

The PR-MOL was administered over sixteen individual sessions, divided into three sessions for pretesting, ten sessions for application of the remediation program for students in the experimental group, and three sessions for post-testing.

To avoid expectation bias, the tendency to interpret the results of the intervention as better or worse according to one’s view of the intervention, the post-testing evaluation was performed by a member of the LIDA Laboratory who was not involved with the pretesting data or the application of the developed program.

The analysis of the results was conducted using the Statistical Package for Social Sciences (SPSS), version 25.0. The Shapiro–Wilk test was conducted to check for normality of the data distribution. Since the results indicated that the data did not follow a normal distribution, the non-parametric Wilcoxon signed-tank test was used to perform intra-group comparisons between two assessment moments, namely, pre- and post-testing. A significance level of 5% (0.050) was adopted. In cases of statistically significant differences, the data were marked with an asterisk in the tables.

## 3. Results

Table 3 and Table 4 show the means, standard deviations, and *p* values for the performance of GI and GII students in the syllabic and phonemic metalinguistic skills tests in PROHMELE compared between pre- and post-testing evaluations.

The Wilcoxon signed-rank test confirmed that there was a statistically significant difference in the performance of GI students in most of the metalinguistic skills tests, except for the identification, syllable number, final phoneme identification, and syllable segmentation tests, compared between the pre- and post-testing evaluations. The superior performance of metaphonological skills after the completion of the remediation program demonstrates an improvement in these skills, which are considered predictors of reading development and involve the participation of working memory to manipulate sounds and syllables. For the GII students, there were no statistically significant differences between the two evaluation times, indicating that the results were similar.

Table 5 shows the means, standard deviations, and *p* values for the performance of GI and GII students in the classification of decoding rules D1, D2, and D4 in the PROHMELE real-word reading test compared between the pre- and post-testing evaluations.

The Wilcoxon signed-rank test showed that there was a statistically significant difference in the classification of decoding rules D1 and D2 by GI students between the two evaluation times, showing an improvement in word decoding and the alphabetic principle after the completion of the remediation program based on the increase in the number of correct answers. However, there was no statistically significant difference in the reading time of real words.

For GII students, there were no statistically significant differences between the two evaluation times. However, there was a statistically significant difference in the total reading time of real words between the two evaluation times.

Table 6 shows the means, standard deviations, and *p* values for the performance of GI and GII students in the classification of decoding rule D1 in the pseudoword reading test of PROHMELE compared between the pre- and post-testing evaluations.

The Wilcoxon signed-rank test indicated a statistically significant difference in the classification of decoding rule D1 in the pseudoword reading test by GI students between the two evaluation times, demonstrating an improvement in storage and manipulation of the phonological loop of working memory. There was no statistically significant difference in the performance of GII students between the two evaluation times.

Table 7 shows the means, standard deviations, and *p* values for the performance of GI and GII students in the Repetition of Digit Sequence in Indirect Order test of NEUPSILIN-Inf compared between the pre- and post-testing evaluations.

The Wilcoxon signed-rank test verified that there was a statistically significant difference in the performance of GI students in the Repetition of Digit Sequence in Indirect Order test between the two evaluation times, which demonstrates an increase in the storage and management capacity of working memory. For GII students, there was no statistically significant difference in performance between the two evaluation times.

Table 8 shows the means, standard deviations, and *p* values for the performance of GI and GII students in the Pseudoword Span test of the NEUPSILIN-Inf compared between the pre- and post-testing evaluations.

The Wilcoxon signed-rank test verified a statistically significant difference in the performance on the Pseudoword Span test in GI students but not in GII students between the two evaluation times. The increase in the number of right answers by the GI group also demonstrates an improvement in the storage and manipulation of information by the phonological loop of the working memory.

Table 9 presents the means, standard deviations, and *p* values for the performance of GI and GII students in the Corsi block test of NEUPSILIN-Inf compared between the pre- and post-testing evaluations.

The Wilcoxon signed-rank test verified that there was a statistically significant difference in the performance of GI students in the Corsi block test between the pre- and post-testing evaluations. This statistical difference shows an increase in the visuospatial working memory storage and management capacity of the GI students. In GII students, there was no statistically significant difference in the performance on the Corsi block test between the pre- and post-testing evaluations.

## 4. Discussion

In phase 2 of this study, the application of PR-MOL in a pilot study of students with learning difficulties demonstrated the applicability of the program, with superior performance observed in the metalinguistic skills tests for the manipulation of both syllables and phonemes, the WM skills test, and the word and pseudoword reading tests.

The improvement in the GI students’ performance after completing the PR-MOL revealed a change in the phonological decoding component of phonological working memory and the achievement of the Portuguese alphabetic principle. The results support the hypothesis that systematic work with working memory produces more effective results when the training of this type of memory is linked to reading tasks. However, when analysing the effectiveness of phonological and working memory interventions in the development of reading skills, it is important to consider the specificities of each language, since the language in which the training is carried out and its level of orthographic consistency can influence the results found. In the case of Brazilian Portuguese, which is an alphabetic language with moderate orthographic consistency, the development of phonological processing skills is essential for learning to read.

The results of the study also reinforce the fundamental role that working memory can play in the initial period of reading acquisition in schoolchildren, as highlighted in previous studies [51,52,53] and demonstrated by the performance of the students who participated in this study in the pretesting reading tests. As the student establishes decoding and begins to learn the orthographic principle of the language, automatic recognition of words by the lexical route, the role of working memory tends to be reduced and focuses more on more complex cognitive tasks, such as comprehension [54,55].

In the metalinguistic skills tests of PROHMELE, the performance of GI students was better than that of GII students in all the tests that involved the manipulation of syllables and phonemes, namely, addition of syllables and phonemes, subtraction of syllables and phonemes, replacement of syllables and phonemes, and combination of syllables and phonemes. This result suggested that the students developed the phonological representations necessary to activate the generative memory mechanism and trigger the grapheme—phoneme conversion mechanism in reading, corroborating the findings of previous studies [24,35,36,37,38].

The same finding was observed in the reading of real words and pseudowords, in which the students had a greater number of correct answers regarding decoding rule D1 in the post-testing evaluation. This result demonstrates greater mastery of the alphabetic principle of the Portuguese language and decoding, which may indicate an increase in the management and storage capacity of information in the phonological WM generated by the PR-MOL, avoiding overload in this subcomponent. This increase can also be evidenced by the pseudoword repetition tests of PROHMELE and the Pseudoword Span test of the NEUPSILIN-Inf, since even with the increase in pseudoword syllables, the students performed the repetition correctly. The same result was observed in the tasks of the remediation program that involved the repetition of words and pseudowords. During the sessions, as the number of words and the length of the sequences increased, the students had a greater number of correct answers.

In addition to the increase in the performance of the students regarding the D1 rule, students who underwent the PR-MOL exhibited a greater number of correct answers than the control students in relation to the context-dependent decoding rule D2, although these rules were not directly stimulated during the program. These results indicate the expansion of the orthographic mental lexicon of these students generated from the expansion of visuospatial WM, as evidenced by the results of the Corsi block test; these results can also be explained by the judicious selection of the active elements of the stimuli used in the program (real words) and the strategies by which they were presented. Therefore, from the frequent visual exposure to writing during the PR-MOL, with each attempt to decode words, the students stored more orthographic information of the words in WM; the information was subsequently transferred to long-term memory and recognised by the lexical route rather than the phonological route [56].

Despite the positive gains in metaphonological skills of the students who underwent the PR-MOL and increases in correct answers to the real word and pseudoword reading tests and in the ability to store and manage phonological and visuospatial WM, a decrease in reading time was observed in students who did not undergo the PR-MOL. This result can be explained by the influence of the teaching dynamics offered by the school, as this is a skill that is constantly adjusted in the classroom.

This finding needs to be validated in a population applicability study with a larger sample, potentially including students with learning difficulties who achieved literacy through different methodologies.

Notably, during the administration of the PR-MOL in the pilot study, an increase in the motivation of the students during the intervention was observed, which can be explained by the playful strategy “Assembling the Memory Treasure” developed together with the tasks of the program, resulting in an improvement in the present study.

This motivational effect observed in the administration of the PR-MOL was also observed in studies by St Clair-Thompson et al. [39], Alloway et al. [24], Maehler et al. [17], Lotfi et al. [29], and Mascarello and Mota [40], who also used a playful context to carry out WM training and observed an increase in adherence and performance during the interventions by the students.

This improvement, which is based on motivation, can be attributed to individual factors such as personality and emotional issues involved in this type of intervention, which require greater cognitive effort from students with WM deficits and learning difficulties [57,58].

Additionally, in this playful context, the instructions for performing the tasks were systematised, with a sequence of processes to be performed in all the sessions, which resulted in the acquisition of a cognitive routine, as proposed by Gathercole [59]. As the students completed the sessions, the execution of this routine became more autonomous, prompting the transfer of this learned routine to long-term memory and, consequently, the ability of these students to apply this cognitive routine to other tasks of similar structure, as demonstrated in the post-testing evaluation, which showed improvement in the performance of the metalinguistic tests of reading and WM.

Due to the acquisition of the metacognitive routine by the students, it is suggested that the PR-MOL contributed to the development of self-regulation skills through cognitive strategies, corroborating the study by Beber et al. [60], which states that when learning becomes effective and maintained in long-term memory, the individual’s behaviour is modified.

Thus, it is essential to continue this research by applying the PR-MOL in a population-based study to verify the consistency of the findings from the pilot study. A major limitation of the present study is that the sample consisted of students who achieved literacy through a single method, preventing an analysis of how different teaching approaches may influence the effectiveness of PR-MOL. Additionally, the small sample size may limit the generalisability of the results, as it does not fully capture the variability present in larger and more diverse student populations. Another important factor to consider for future replications of the study is the influence of the linguistic characteristics of the language in which the intervention is being conducted. Furthermore, potential biases in participant selection and the influence of external factors, such as prior exposure to other literacy interventions, should be taken into account in future research. Therefore, to enhance the validity and applicability of the findings, larger-scale studies are recommended.

A remediation program for phonological and visuospatial WM skills associated with the ability to decode reading for students with learning difficulties was developed, and the use of this program in a pilot study resulted in increased performance in the ability and manipulation of WM and reading decoding ability of students with learning difficulties.

Thus, the structured remediation program for WM difficulties demonstrated applicability and can help education professionals intervene in deficits in WM and reading decoding skills presented by students with learning difficulties.

## Figures and Tables

**Table 1:** Tasks contained in the programs for WM.

Phonological WM	Visuospatial Working Memory
Repetition of words in the same order and/or in reverse order; Repetition of pseudowords in the same order and/or in reverse order; Repetition of Digits and colours in the same order and/or in reverse order; Recall of sequences of phonological representations of graphemes and comparisons between them; Naming of figures and digits; Execution of simultaneous tasks to perform in a request order; Memorization of short stories; N-back tasks, in which the recall of one the elements of the presented sequence is requested according its position; Interpretation of sentences and memorization of the last word of each of the sentences.	Immediate repetition of a sequence of visuospatial information in the same order; Tracing of locations presented sequentially by a target stimulus; Replication of the visuospatial path in a maze; Identification and retrieval of hidden objects; Retelling films and stories; Memory games; Mental rotation of letter.

**Table 2:** Modules of the Remediation Program with WM and Reading (RP-WMR).

Phonological Working Memory	Visuospatial Working Memory
(1) *Repetition of words in the same order:* The administrator says a sequence of words, and the student must repeat this sequence in the same order.	(1) *Indication of pictures in the order shown*: The administrator places pictures on the table and then shows a sequence of pictures to the student. The student must indicate the pictures in the same order presented by the administrator.
(2) *Repetition of words in reverse order:* The administrator says a sequence of words, and the student must repeat this sequence in reverse order.	(2) *Indication of pictures in reverse order:* The administrator places pictures on the table and then shows a sequence of pictures to the student. The student must indicate the pictures in the reverse order of that presented by the administrator.
(3) *Repetition of pseudowords in the same order:* The administrator says a sequence of pseudowords and the student must repeat this sequence in the same order.	(3) *Indication of words in the order shown*: The administrator places some words on the table and then shows a sequence of them to the student. The student must indicate the words in the same order presented by the administrator.
(4) *Repetition of pseudowords in reverse order:* The administrator says a sequence of pseudowords, and the student must repeat this sequence in reverse order.	(4) *Indication of words in reverse order*: The administrator places some words on the table and then shows a sequence of them to the student. The student must indicate the words in the reverse order of that presented by the administrator.
(5) *Indication of pictures in the same order as the sequence spoken by the* administrator: The administrator displays some pictures on the table and then says a sequence of words. The student must indicate the pictures in the same order as the spoken sequence.	(5) *Remembering and reproducing the route taken by the administrator in a dot matrix and the locations along the route:* The administrator traces a predefined route on a city map, starting from a fixed point and passing through 1, 2 or 3 places. At the end of each course, the student must reproduce it in the same order.
(6) *Indication of pictures in reverse order from the sequence spoken by the* administrator: The administrator displays some pictures on the table and then says a sequence of words. The student must indicate the pictures in reverse order from the spoken sequence.	

**Table 3:** Distribution of the means, standard deviations, and *p* values for the performance of GI and GII students in the syllabic metalinguistic skills tests in PROHMELE compared between the pre- and post-testing evaluations.

G	Skill	No.	Pre-M (SD)	Post-M (SD)	*p* Value
GI	ISI	11	9.82 (0.41)	9.91 (0.30)	0.564
GII	ISI	10	9.70 (0.48)	9.40 (1.08)	0.276
GI	FSI	11	6.55 (1.04)	9.00 (1.00)	0.003 *
GII	FSI	10	7.00 (2.26)	7.00 (2.31)	>0.999
GI	MSI	11	6.36 (1.80)	8.91 (1.58)	0.005 *
GII	MSI	10	8.20 (1.87)	7.40 (2.95)	0.266
GI	SYLSUBT	11	6.27 (2.41)	8.82 (1.17)	0.005 *
GII	SYLSUBT	10	3.80 (3.39)	5.50 (3.63)	0.258
GI	SYLAD	11	5.36 (3.17)	9.82 (0.41)	0.005 *
GII	SYLAD	10	4.70 (3.30)	5.10 (4.23)	0.671
GI	SYLSUBS	11	4.73 (1.95)	8.18 (1.40)	0.003 *
GII	SYLSUBS	10	2.70 (2.95)	2.80 (3.01)	0.892
GI	SYLCOMB	11	4.55 (1.04)	8.36 (1.69)	0.004 *
GII	SYLCOMB	10	2.20 (1.75)	3.10 (3.57)	0.347
GI	SYLSEG	11	9.55 (0.69)	10.00 (0.00)	0.059
GII	SYLSEG	10	9.00 (1.56)	8.90 (1.20)	0.705

Legend: * *p* < 0.05, ISI: initial syllable identification, FSI: final syllable identification, MSI: middle syllable identification, SYLSUBT: syllable subtraction, SYLAD: syllable addition, SYLSUBS: syllable substitution, SYLCOMB: syllable combination, SYLSEG: syllable segmentation.

**Table 4:** Distribution of the means, standard deviations, and *p* values for the performance of GI and GII students in the phonemic metalinguistic skills tests in PROHMELE compared between the pre- and post-testing evaluations.

G	Skill	No.	Pre-M (SD)	Post-M (SD)	*p* Value
GI	IPI	11	8.00 (1.18)	9.64 (0.51)	0.007 *
GII	IPI	10	8.40 (1.71)	8.10 (1.60)	0.595
GI	FPI	11	7.36 (1.21)	8.18 (1.08)	0.204
GII	FPI	10	7.50 (1.84)	8.00 (1.33)	0.339
GI	MPI	11	6.18 (1.94)	8.73 (1.01)	0.006 *
GII	MPI	10	6.80 (2.25)	7.40 (1.71)	0.395
GI	PHOSUBT	11	3.27 (2.28)	8.09 (1.70)	0.003 *
GII	PHOSUBT	10	2.60 (3.41)	2.60 (3.31)	>0.999
GI	PHOAD	11	3.82 (2.68)	7.91 (1.70)	0.003 *
GII	PHOAD	10	4.30 (3.34)	3.80 (2.97)	0.356
GI	PHOSUBS	11	1.82 (1.60)	7.00 (2.10)	0.003 *
GII	PHOSUBS	10	1.70 (2.87)	0.90 (1.52)	0.279
GI	PHOCOMB	11	3.27 (2.05)	7.55 (2.51)	0.003 *
GII	PHOCOMB	10	2.20 (2.94)	0.30 (0.48)	0.078
GI	PHOSEG	11	1.45 (1.81)	7.91 (2.84)	0.005 *
GII	PHOSEG	10	0.00 (0.00)	0.00 (0.00)	>0.999

Legend: * *p* < 0.05, IPI: initial phoneme identification; FPI: final phoneme identification, MPI: middle phoneme identification, PHOSUBT: phoneme subtraction, PHOAD: phoneme addition, PHOSUBS: phoneme substitution, PHOCOMB: phoneme combination, PHOSEG: phoneme segmentation.

**Table 5:** Distribution of the means, standard deviations, and *p* values for the performance of GI and GII students in the classification of decoding rules D1, D2, and D4 compared between the pre- and post-testing evaluations.

G	Decoding Rules	n	Pre-M (SD)	Post-M (SD)	*p* Value
GI	D1	11	105.82 (68.74)	162.00 (8.38)	0.003 *
GII	D1	10	92.40 (80.21)	94.80 (81.68)	0.496
GI	D2	11	266.45 (171.77)	400.00 (17.31)	0.003 *
GII	D2	10	230.30 (200.75)	238.20 (205.52)	0.115
GI	D4	11	0.00 (0.00)	0.73 (1.27)	0.066
GII	D4	10	0.40 (0.70)	0.33 (0.50)	0.317
GI	time	11	8.60 (8.51)	8.53 (4.88)	0.534
GII	time	10	4.57 (5.00)	2.44 (2.59)	0.028 *

Legend: * *p* < 0.05.

**Table 6:** Distribution of the means, standard deviations, and *p* values for the performance of GI and GII students in the classification of decoding rule D1 in the PROHMELE pseudoword reading test compared between the pre- and post-testing evaluations.

G	Decoding Rule	n	Pre-M (SD)	Post-M (SD)	*p* Value
GI	ROP_D1	11	23.73 (10.40)	36.00 (4.05)	0.003 *
GII	ROP_D1	10	17.20 (15.96)	17.70 (15.65)	0.395

Legend: * *p* < 0.05, ROP: reading of pseudowords.

**Table 7:** Distribution of the means, standard deviations, and *p* values for the performance of GI and GII students in the Repetition of Digit Sequence in Indirect Order test of NEUPSILIN-Inf compared between the pre- and post-testing evaluations.

G	Pair of Variables	n	Pre-M (SD)	Post-M (SD)	*p* Value
GI	RDS_IO	11	13.64 (2.80)	16.45 (3.24)	0.032 *
GII	RDS_IO	10	10.20 (5.31)	12.30 (8.07)	0.372

Legend: * *p* < 0.05; RDS_IO: Repetition of Digit Sequence in Indirect Order.

**Table 8:** Distribution of the means, standard deviations, and *p* values for the performance of GI and GII students in the Pseudoword Span test of NEUPSILIN-Inf compared between the pre- and post-testing evaluations.

G	Pair of Variables	n	Pre-M (SD)	Post-M (SD)	*p* Value
GI	SPAN_PW	11	9.64 (2.50)	12.82 (3.13)	0.005 *
GII	SPAN_PW	10	7.50 (3.06)	8.80 (2.82)	0.176

Legend: * *p* < 0.05; SPAN_PW: span of pseudowords.

**Table 9:** Distribution of the means, standard deviations, and *p* values for the performance of GI and GII students in the Corsi block test of NEUPSILIN-Inf compared between the pre- and post-testing evaluations.

G	Pair of Variables	n	Pre-M (SD)	Post-M (SD)	*p* Value
GI	CB	11	20.36 (5.77)	24.00 (4.31)	0.005 *
GII	CB	10	15.60 (6.15)	13.20 (7.10)	0.057

Legend: * *p* < 0.05, CB: Corsi block.

## Data Availability

The data presented in this study are available on request from the corresponding author due to participant privacy and ethical reasons.

## References

[1] Beneventi H., Tønnessen F.E., Ersland L., Hugdahl K. (2010). Working memory deficit in dyslexia: Behavioral and fMRI evidence. Int. J. Neurosci..

[2] Gathercole S.E., Pickering S.J. (2000). Working memory deficits in children with low achievements in the national curriculum at 7 years of age. Br. J. Educ. Psychol..

[3] Alloway T.P., Gathercole S.E., Pickering S.J. (2006). Verbal and visuospatial short-term and working memory in children: Are they separable?. Child Dev..

[4] Baddeley A.D., Hitch G. (1974). Working Memory. Psychol. Learn. Motiv..

[5] Logie R.H. (1995). Visuo-Spatial Working Memory.

[6] Gathercole S.E., Pickering S.J., Ambridge B., Wearing H. (2004). The structure of working memory from 4 to 15 years of age. Dev. Psychol..

[7] Alloway T.P., Copello E. (2013). Working memory: The what, the why, and the how. Aust. Educ. Dev. Psychol..

[8] Miyake A., Friedman N.P., Emerson M.J., Witzki A.H., Howerter A., Wager T.D. (2000). The unity and diversity of executive functions and their contributions to complex “frontal lobe” tasks: A latent variable analysis. Cogn. Psychol..

[9] Gathercole S.E., Pickering S.J., Knight C., Stegmann Z. (2004). Working memory skills and educational attainment: Evidence from national curriculum assessments at 7 and 14 years of age. Appl. Cogn. Psychol..

[10] Cockcroft K., Alloway T. (2012). Phonological awareness and working memory: Comparisons between South African and British children. S. Afr. Linguist. Appl. Lang. Stud..

[11] O’Shaughnessy T.E., Swanson H.L. (2000). A comparison of two reading interventions for children with reading disabilities. J. Learn. Disabil..

[12] Daneman M. (1991). Working memory as a predictor of verbal fluency. J. Psycholinguist. Res..

[13] Chandler P., Sweller J. (1991). Cognitive load theory and the format of instruction. Cogn. Instr..

[14] Sweller J. (1994). Cognitive load theory, learning difficulty, and instructional design. Learn. Instr..

[15] Evans J.S.B.T., Stanovich K.E. (2013). Dual-process theories of higher cognition: Advancing the debate. Perspect. Psychol. Sci..

[16] Maehler C., Schuchardt K. (2016). Working memory in children with specific learning disorders and/or attention deficits. Learn. Individ. Differ..

[17] Maehler C., Joerns C., Schuchardt K. (2019). Training working memory of children with and without dyslexia. Children.

[18] Dias N.M., Seabra A.G. (2017). School performance at the end of elementary school: Contributions of intelligence, language, and executive functions. Estud. Psicol..

[19] Novaes C.B., Zuanetti P.A., Fukuda M.T.H. (2019). Effects of working memory intervention on students with reading comprehension difficulties. Rev. CEFAC.

[20] Alloway T.P., Gathercole S.E., Kirkwood H., Elliott J. (2009). The cognitive and behavioral characteristics of children with low working memory. Child Dev..

[21] Medina G.B.K., Guimarães S.R.K. (2021). Reading in developmental dyslexia: The role of phonemic awareness and executive functions. Estud. Psicol..

[22] de Carvalho C.A.F., Kida A.D.S.B., Capellini S.A., de Avila C.R.B. (2014). Phonological working memory and reading in students with dyslexia. Front. Psychol..

[23] Silva C., Capellini S.A. (2013). Desempenho de escolares com e sem transtorno de aprendizagem em leitura, escrita, consciência fonológica, velocidade de processamento e memória de trabalho fonológica. Rev. Psicopedag..

[24] Alloway T.P., Bibile V., Lau G. (2013). Erratum: Computerized working memory training: Can it lead to gains in cognitive skills in students? (Computers in Human Behavior (2012) 29 (632–638)). Comput. Hum. Behav..

[25] Song J., MacQuarrie S., Hennessey A. (2023). Working memory training: Mechanisms, challenges and implications for the classroom. Front. Educ..

[26] Tan A.S.L., Lau R.C., Anderson P.J., Gathercole S., Bellgrove M.A., Wiley J.F., Spencer-Smith M.M. (2024). Exploring working memory capacity and efficiency processes to understand working memory training outcomes in primary school children. J. Cogn..

[27] Peijnenborgh J.C.A.W., Hurks P.M., Aldenkamp A.P., Vles J.S.H., Hendriksen J.G.M. (2016). Efficacy of working memory training in children and adolescents with learning disabilities: A review study and meta-analysis. Neuropsychol. Rehabil..

[28] Rodas J.A., Asimakopoulou A.A., Greene C.M. (2024). Can we enhance working memory? Bias and effectiveness in cognitive training studies. Psychon. Bull. Rev..

[29] Lotfi S., Rostami R., Shokoohi-Yekta M., Ward R.T., Motamed-Yeganeh N., Mathew A.S., Lee H.-J. (2020). Effects of computerized cognitive training for children with dyslexia: An ERP study. J. Neurolinguistics.

[30] Peng P., Fuchs D. (2017). A Meta-Analysis of Working Memory Deficits in Children with Learning Difficulties: Is There a Difference Between Verbal Domain and Numerical Domain?. J. Learn. Disabil..

[31] Dahlin K.I.E. (2011). Effects of Working Memory Training on Reading in Children with Special Needs. Read. Writ..

[32] Shipstead Z., Redick T.S., Engle R.W. (2012). Is Working Memory Training Effective?. Psychol. Bull..

[33] Wang S., Gathercole S.E., Ren X., Zhou H. (2014). Working Memory Training in Chinese Children with Low Working Memory: Transfer and Maintenance Effects. Child Dev..

[34] Barnes M.A., Huber J., Johnston A.M., Dennis M., Tannock R., Schachar R. (2016). Attention and Executive Functions in Children with Spina Bifida Myelomeningocele. Child. Neuropsychol..

[35] Chen X., Ye M., Chang L., Chen W., Zhou R. (2018). Effect of working memory updating training on retrieving symptoms of children with learning disabilities. J. Learn. Disabil..

[36] Dehghani Y., Moradi N.A. (2019). The effectiveness of working memory training on inhibition andreading performance of students with specific learning disabilities (dyslexia). Neuropsychology.

[37] Sharifi S., Rezaei S. (2018). The effectiveness of working memory training on reading difficulties among students with reading disorder. Iran. J. Learn. Mem..

[38] Yang J., Peng J., Zhang D., Zheng L., Mo L. (2017). Specific effects of working memory training on the reading skills of Chinese children with developmental dyslexia. PLoS ONE.

[39] St Clair-Thompson H., Stevens R., Hunt A., Bolder E. (2010). Improving children’s working memory and classroom performance. Educ. Psychol..

[40] Mascarello L.J., Mota M.B. (2019). Efeitos de uma intervenção voltada para a memória de trabalho de crianças em processo de alfabetização. ReVEL.

[41] Luo Y., Wang J., Wu H.R., Zhu D.M., Zhang Y. (2013). Working-memory training improves developmental dyslexia in Chinese children. Neural Regen. Res..

[42] Mahdavi A., Taghizadeh M.E., Isazadeh S., Kazem Nia K., Vojdani S., Hosseini S.N. (2015). Effectiveness of Education of Working Memory Strategies on Improvement of Reading Performance and Reduction of Depression in Children with Dyslexia. Mediterr. J. Soc. Sci..

[43] Sheykholeslami A., Bakhshayesh A.R., Barzegar-Bafrooei K., Vajihe M.A. (2017). The Effectiveness of Working Memory Training on Reading Performance and Memory Capacity of Students with Reading Disability. J. Clin. Psychol..

[44] Kakabaraee K., Afsharinia K., Bostan N., Arefi M. (2015). Investigating Effect of Working Memory Training on Reading Performance in Students with Reading Disorders. Int. J. Innov. Res. Educ. Sci..

[45] Rahimipour T., Ghazanfari F., Ghadampour E. (2018). The effectiveness of working memory strategies training on improvement of reading performance in dyslexic students. Knowl. Res. Appl. Psychol..

[46] Alloway T.P. (2012). Can interactive working memory training improve learning?. J. Interact. Learn. Res..

[47] Oliveira A.M., Capellini S.A. (2018). E-Leitura II: Banco de Palavras de Alta, Média e Baixa Frequência para Escolares do Ensino Fundamental I.

[48] Alloway T.C., Passolunghi M.C. (2011). The relationship between working memory, IQ and mathematical skills in children. Learn. Individ. Differ..

[49] Cunha V.L.O., Capellini S.A. (2022). Provas de Habilidades Metalinguísticas e de Leitura: PROHMELE.

[50] Salles J.F., Fonseca R.P., Parente MA M.P., Miranda M.C., Rodrigues C.C., Mello C.B., Barbosa T. (2016). Instrumento de Avaliação Neuropsicológica Breve Infantil: NEUPSILIN-Inf.

[51] Banales E., Kohnen S., McArthur G. (2015). Can verbal working memory training improve reading?. Cogn. Neuropsychol..

[52] Dehn M.J. (2011). Working Memory and Academic Learning: Assessment and Intervention.

[53] Dias N., Seabra A.G., Mackenzie U.P. (2015). Funções executivas: Desenvolvimento e intervenção. Temas Sobre Desenvolv..

[54] Spencer J.P. (2020). The development of working memory. Curr. Dir. Psychol. Sci..

[55] Unsworth N., McMillan B.D. (2013). Mind wandering and reading comprehension: Examining the roles of working memory capacity, interest, motivation, and topic experience. J. Exp. Psychol. Learn. Mem. Cogn..

[56] Barbosa P.M.F., Bernardes N.G.B., Misorelli M.I., Chiappetta A.L.D.M.L. (2010). Relação da memória visual com o desempenho ortográfico de crianças de 2nd e 3rd séries do ensino fundamental. Rev. CEFAC.

[57] Titz C., Karbach J. (2014). Working memory and executive functions: Effects of training on academic achievement. Psychol. Res..

[58] Spencer-Smith M., Weinman A., Quach J., Pascoe L., Mensah F., Wake M., Roberts G., Anderson P.J. (2023). Grit and working memory training outcomes for children with low working memory. Acta Paediatr..

[59] Gathercole S.E., Dunning D.L., Holmes J., Norris D. (2019). Working Memory involves learning new skills. J. Mem. Lang..

[60] Beber B., Silva E., Bonfiglio S. (2014). Metacognição como processo da aprendizagem. Rev. Psicopedag..

